# *Actinidia chinensis* Planch Root extracts trigger ferroptosis in colorectal cancer via the p53/SLC7A11/GPX4 axis

**DOI:** 10.3389/fphar.2026.1724983

**Published:** 2026-01-14

**Authors:** Chenyang Ma, Ruixiu Chen, Hangxuan Wang, Kaijie Qiu, Qing Xia, Shaohui Yang, Jianan Wang, Zisheng Cheng, Wei Cui, Jun Lu, Shiwei Duan

**Affiliations:** 1 Department of Colorectal Surgery, Ningbo Medical Center Lihuili Hospital, Ningbo, Zhejiang, China; 2 Key Laboratory of Novel Targets and Drug Study for Neural Repair of Zhejiang Province, School of Medicine, Hangzhou City University, Hangzhou, Zhejiang, China; 3 Department of Hepatobiliary and Pancreatic Surgery, Ningbo Medical Center Lihuili Hospital, Ningbo, Zhejiang, China; 4 College of pharmacy, Zhejiang University of Technology, Hangzhou, Zhejiang, China

**Keywords:** acRoots, cell proliferation, CRC, ferroptosis, p53

## Abstract

**Purpose:**

Colorectal cancer (CRC) remains a leading cause of cancer-related mortality worldwide. *Actinidia chinensis* Planch Root extracts (acRoots), a traditional Chinese medicine (TCM), possess recognized anticancer properties, but their efficacy and mechanism in Colorectal cancer are not fully understood. This study investigates the role of acRoots in suppressing Colorectal cancer progression, with a specific focus on its potential to induce ferroptosis, a form of iron-dependent cell death.

**Methods:**

The anti-tumor effects of acRoots were evaluated in human Colorectal cancer cell lines (HCT-15, CW-2) using Cell Counting Kit-8 (CCK-8), colony formation, wound healing, and Transwell assays. Cell death was analyzed by Annexin V-FITC/PI flow cytometry. Mechanisms were probed by measuring reactive oxygen species (ROS), glutathione (GSH) levels, malondialdehyde (MDA) levels, and performing qRT-PCR and Western blot for ferroptosis-related markers (SLC7A11, GPX4, p53). The ferroptosis inhibitor Ferrostatin-1 (Fer-1) was used in rescue experiments. The *in vivo* antitumor efficacy was assessed in HCT-15 xenograft models in nude mice treated orally with acRoots (150 mg/kg/day) for 14 days.

**Results:**

The acRoots significantly and dose-dependently inhibits the viability, proliferation, migration, and invasion of colorectal cancer cells. Concurrently, acRoots effectively induces ferroptosis in these cells, characterized by intracellular reactive oxygen species (ROS) accumulation, glutathione (GSH) depletion, and a significant increase in the lipid peroxidation product malondialdehyde (MDA). These ferroptosis-related phenotypes can be reversed by the ferroptosis-specific inhibitor Ferrostatin-1 (Fer-1). Mechanistically, acRoots upregulates the expression of the tumor suppressor gene p53, subsequently downregulating the expression levels of key ferroptosis regulators SLC7A11 and GPX4. Furthermore, pretreatment with Fer-1 effectively reverses acRoots-induced cytotoxicity and ROS accumulation. In an HCT-15 xenograft mouse model, oral administration of acRoots (150 mg/kg/day) significantly inhibited tumor growth, reduced intratumoral GSH levels, and no obvious toxicity was observed.

**Conclusion:**

Our findings demonstrate that acRoots exerts potent anti-Colorectal cancer effects by inhibiting malignant phenotypes and inducing ferroptosis. This ferroptosis is mediated, at least in part, through the p53-dependent downregulation of the SLC7A11/GPX4 axis. These results position acRoots as a promising therapeutic candidate and a novel natural ferroptosis inducer for Colorectal cancer treatment, warranting further clinical investigation.

## 1 Introduction

Colorectal cancer represents a major global health challenge. According to the latest estimates from the International Agency for Research on Cancer (IARC), there were over 1.9 million new cases and approximately 904,000 deaths from Colorectal cancer in 2022, accounting for nearly one-tenth of the global cancer burden (Bray et al., 2024). Colorectal cancer currently ranks third in incidence and second in mortality among all malignancies worldwide (Bray et al., 2024). Alarmingly, projections indicate that the global incidence of Colorectal cancer is poised to more than double by 2035 (Hossain et al., 2022), underscoring an urgent need for efficient therapeutic strategies.

The current treatment for Colorectal cancer encompasses surgery, radiotherapy, chemotherapy, targeted therapy, and immunotherapy (Kanani et al., 2021). Conventional chemotherapeutic agents, such as 5-fluorouracil, tegafur-uracil, and S-1, remain cornerstone treatments (García-Alfonso et al., 2021). However, their efficacy is often hampered by severe side effects and systemic toxicity, including myelosuppression, gastrointestinal disturbances, and fatigue (García-Alfonso et al., 2021; Shinji et al., 2022). Consequently, the pursuit of alternative anticancer agents with enhanced efficacy and reduced adverse effects remains a paramount objective in oncology research.

In this context, TCM has garnered increasing attention for its potential in cancer management, offering unique advantages such as multi-target effects, low toxicity, and the ability to mitigate chemotherapy-induced side effects and inhibit metastasis (Zou et al., 2024). Notably, TCM has shown particular promise in treating digestive diseases—especially Colorectal cancer. Accumulating clinical and preclinical evidence supports its value in Colorectal cancer therapy: TCM formulations can reshape the anti-tumor microenvironment by regulating gut microbiota homeostasis and intestinal mucosal barrier function (Huang G. et al., 2022), while TCM-derived compounds often synergize with chemotherapeutics by targeting key signaling pathways involved in Colorectal cancer cell proliferation and survival (Xia et al., 2022). *Actinidia chinensis* Planch Root extracts (acRoots) are a prominent TCM with documented anticancer properties. Our previous phytochemical analysis of acRoots indicates that it contains a variety of bioactive components, which may collectively contribute to its pharmacological activity, including phenolics, flavonoids, terpenoids, and other compounds (Qiu et al., 2023). Notably, some of these constituents, such as chlorogenic acid and swertiamarin, have been independently reported to exert anti-cancer effects through mechanisms such as inducing apoptosis and arresting the cell cycle (Perkasa et al., 2025; Gao et al., 2020). The complex combination of these bioactive molecules in acRoots likely forms the material basis for the multi-target anti-cancer properties explored in this study. A growing body of evidence from *in vitro* and *in vivo* studies demonstrates that acRoots can inhibit proliferation and induce apoptosis in a variety of cancer cells, including those from gastric, leukemia, colon, liver, and lung cancers (Huo et al., 2013; Cheng et al., 2015; Song et al., 2014; Lv et al., 2018). Furthermore, the ethanol extract of acRoots has been shown to suppress tumor angiogenesis and significantly impede tumor growth in colon cancer models (Hu et al., 2021; Yuan et al., 2021). Huangqin Decoction is a well-known traditional formula. It contains key botanicals such as *Scutellaria baicalensis* (Huangqin), *Coptis chinensis* (Huanglian), and *Gardenia jasminoides* (Zhizi). These components have documented anti- Colorectal cancer activities. The decoction has shown notable clinical efficacy as an adjuvant therapy for colon cancer (Chen et al., 2018), and a synergistic combination of acRoots and *Scutellaria baicalensis* has demonstrated effectiveness against Colorectal cancer (Huang J. et al., 2022). Despite these promising observations, the precise molecular mechanisms underpinning the anti-Colorectal cancer activity of acRoots warrant further in-depth investigation. Despite these promising observations, the precise molecular mechanisms underpinning the anti-Colorectal cancer activity of acRoots remain unknown.

Recently, ferroptosis has emerged as a compelling frontier in cancer biology and therapy. This novel form of regulated cell death is distinct from apoptosis and is characterized by iron-dependent accumulation of lethal lipid hydroperoxides (Dixon et al., 2012; Stockwell et al., 2017). The core defense system against ferroptosis revolves around GSH and glutathione peroxidase 4 (GPX4), which work in concert to reduce lipid peroxides to non-toxic alcohols (Ursini and Maiorino, 2020). The solute carrier family 7 member 11 (SLC7A11), a key metabolite of the cystine/glutamate antiporter system Xc-, is critical for the synthesis of GSH, and its inhibition is a recognized mechanism for inducing ferroptosis (Koppula et al., 2021). Notably, several agents, including Tagitinin C, Cetuximab, and Elesclomol, have been shown to trigger ferroptosis in Colorectal cancer cells (Wei et al., 2021; Yang et al., 2021; Gao et al., 2021), highlighting its therapeutic relevance. Intriguingly, network pharmacology screening suggests that acRoots may interact with multiple functional targets for colon cancer treatment, with a notable association with the tumor protein p53. p53, a master tumor suppressor, can induce ferroptosis by transcriptionally repressing SLC7A11, thereby disrupting the cellular redox balance (Xie et al., 2017).

However, the specific role of acRoots in modulating ferroptosis and the potential involvement of the p53/SLC7A11 axis in Colorectal cancer remain entirely unexplored. Therefore, this study aims to systematically investigate whether acRoots suppresses Colorectal cancer by inducing ferroptosis and to elucidate the underlying molecular mechanism, with a specific focus on the role of p53 and its downstream targets, SLC7A11 and GPX4.

## 2 Materials and methods

### 2.1 Preparation and chemical characterization of acRoots

The preparation process of the acRoots used in this study was consistent with that described in our team’s previously published research on anti-hepatocellular carcinoma activity (Qiu et al., 2023), ensuring comparability of the pharmacologically active substance basis across different studies. The complete preparation procedure and chemical characterization information are as follows.

#### 2.1.1 Plant material source and extraction process

The herbal material used was the dried root of *Actinidia chinensis* Planch. The specific extraction procedure was as follows: 100 g of the dried crude drug was weighed, soaked in 300 mL of purified water, and then subjected to reflux decoction for 45 min. The decoction was filtered, and the filtrate was concentrated in a 60 °C water bath to a final volume of 50 mL, yielding an aqueous extract with a herb-to-extract concentration ratio of 2:1 (w/v, meaning 1 mL of the concentrate is equivalent to 2 g of crude drug). This concentrate was frozen at −80 °C for 24 h and then lyophilized using a Martin Christ freeze dryer (Osterode, Germany). The resulting dried powder was stored in a desiccator at room temperature for subsequent use.

The acRoots used in this study were consistent with those reported in our team’s previously published anti-hepatocellular carcinoma research (Qiu et al., 2023) in terms of raw material batch, preparation process, and chemical characterization. That study had already performed systematic chemical composition identification on the same batch of acRoots using UPLC-Triple-TOF/MS technology (main components are listed in Supplementary Table S1; Supplementary Figure S1), thus ensuring the reliability of the pharmacologically active substance basis in this experiment.

#### 2.1.2 Chemical composition analysis

As this study focuses on the preliminary exploration of pharmacological mechanisms, the chemical composition of acRoots was analyzed using Ultra-Performance Liquid Chromatography coupled with Triple Quadrupole Time-of-Flight Mass Spectrometry (UPLC-Triple-TOF/MS). This high-resolution technique has been successfully applied for the qualitative and relative quantitative analysis of the same batch of acRoots in our previous work (Qiu et al., 2023).

The main chemical components identified in acRoots are listed in Supplementary Table S1. A total of 24 compounds were identified, belonging to categories such as flavonoids, terpenoids, organic acids, phenols, glycosides, coumarins, and phenylpropanoids. Their combined relative peak area accounts for 15.08% of the total detected peaks. The UPLC-Triple-TOF/MS base peak chromatogram of acRoots is provided in Supplementary Figure S1, which visually presents the chromatographic separation profile of its chemical constituents.

### 2.2 Preparation of acRoots extract

One hundred grams of dried acRoots were extracted with 300 mL of pure water by refluxing for 45 min. The resulting decoction was filtered, and the filtrate was concentrated to a final volume of 50 mL using a water bath at 60 °C, yielding a concentrated solution with a concentration of 2 g/mL. An aliquot of this solution was frozen at −80 °C for 24 h and then lyophilized using a Martin Christ freeze dryer (Osterode, Germany) to obtain a dry powder. For cell culture experiments, the powder was reconstituted in distilled water to create a 100 mg/mL stock solution, which was stored at 4 °C. Working concentrations were prepared by diluting the stock solution in the complete cell culture medium immediately before use.

### 2.3 Cell culture

Human Colorectal cancer cell lines HCT-15 and CW-2 (iCell Bioscience Inc., Shanghai, China) were cultured in RPMI-1640 medium (Nanjing BioChannel Biotechnology Co., Ltd.), supplemented with 10% FBS (Nanjing BioChannel Biotechnology Co., Ltd.). All cells were maintained at 37 °C in a humidified incubator with 5% CO_2_.

### 2.4 Cell viability assay

Cell viability was assessed using the CCK-8 (MedChemExpress) (Ishiyama et al., 1996). Cells were seeded in 96-well plates at a density of 5 × 10^3^ cells per well. After 24 and 48 h of treatment with acRoots, 10 µL of CCK-8 solution was added to each well. Following a 2-h incubation at 37 °C, the absorbance at 450 nm was measured using a microplate reader (Thermo Fisher Scientific, United States).

### 2.5 Colony formation assay

Cells were seeded into 6-well plates at a low density of 200 cells per well. After 24 h of attachment, they were treated with various concentrations of acRoots for 24 h. The medium was then replaced with a drug-free medium, and the cells were cultured for an additional 2 weeks. The resulting colonies were fixed with methanol, stained with 0.05% crystal violet, and counted (Franken et al., 2006).

### 2.6 Wound healing assay

Cells were seeded into 6-well plates and grown to confluence. A linear wound was created in the cell monolayer using a 200 µL sterile pipette tip. After washing to remove detached cells, the cells were treated with various concentrations of acRoots solution. Images of the wound area were captured at 0 h and 19 h post-scratching using a microscope, and the migration rate was calculated (Liang et al., 2007).

### 2.7 Cell death analysis by flow cytometry

The rate of cell death was determined using an Annexin V-FITC/PI apoptosis detection kit (Rieger et al., 2011). Briefly, HCT-15 and CW-2 cells were treated with acRoots for 24 h, harvested, and washed with phosphate-buffered saline (PBS). The cells were then resuspended in 300 µL of binding buffer and stained with Annexin V-FITC and propidium iodide (PI) for 20 min at room temperature in the dark. The stained cells were analyzed immediately using a BD FACSCalibur flow cytometer (BD Biosciences, United States).

### 2.8 RNA extraction and quantitative Real-Time PCR (qRT-PCR)

Total RNA was extracted from cells using Trizol reagent (Invitrogen, United States) according to the manufacturer’s instructions. cDNA was synthesized from 1 µg of total RNA using a reverse transcription kit. qRT-PCR was performed using SYBR Green Master Mix (ABclonal Technology Co., Ltd.) on a LightCycler 480 II Real-Time PCR System (Roche, Switzerland). The relative mRNA expression levels were calculated using the 2^−ΔΔCt^ method (Livak and Schmittgen, 2001), with Glyceraldehyde-3-phosphate dehydrogenase (GAPDH) as the internal control. The primer sequences used are listed in Supplementary Table S2.

### 2.9 Transwell invasion assay

Cell invasion was assessed using 24-well Transwell chambers (Beijing Lanjieko Technology Co., Ltd.) coated with Matrigel. The assay was performed as previously described (Justus et al., 2023). After treatment with acRoots solution for 24 h, 5 × 10^4^ cells in serum-free medium were seeded into the upper chamber. The lower chamber was filled with 1,640 medium containing 10% FBS as a chemoattractant. After 24 h of incubation, the non-invading cells on the upper surface of the membrane were removed with a cotton swab. The cells that had invaded through the Matrigel to the lower surface were fixed with methanol, stained with 0.1% crystal violet, and counted under a microscope.

### 2.10 Measurement of intracellular ROS

Intracellular ROS levels were detected using the fluorescent probe DCFH-DA (Beyotime, China) (Wang and Joseph, 1999). After treatment with acRoots, cells were incubated with 10 µM DCFH-DA at 37 °C for 20 min in the dark. Subsequently, the cells were washed three times with serum-free medium to remove excess probe. The fluorescence intensity, indicative of ROS levels, was immediately observed and captured using a fluorescence microscope (Nikon, Japan).

### 2.11 GSH assay

Intrac249ellular GSH levels were measured using a GSH assay kit (Beyotime, China) following the manufacturer’s protocol (Rahman et al., 2006). Briefly, treated cells were collected and lysed. After centrifugation at 12,000 × g for 10 min at 4 °C, the supernatant was collected. The protein concentration of the supernatant was determined using a BCA protein assay kit. An aliquot of the supernatant was then mixed with the assay reagents, and the absorbance was measured at 412 nm. The GSH content was normalized to the total protein concentration and expressed relative to the control group.

### 2.12 MDA assay

Intracellular malondialdehyde (MDA) levels were measured using the Elabscience MDA colorimetric assay kit (Elabscience, China), following the manufacturer’s instructions. In brief, treated cells were collected and homogenized in the kit-provided extraction reagent. After centrifugation at 10,000 × g for 10 min at 4 °C, the supernatant was collected. The total protein concentration of the supernatant was determined using a BCA protein assay kit. An aliquot of the supernatant was mixed with freshly prepared working solution and incubated at 100 °C for 40 min. After cooling to room temperature, the mixture was centrifuged at 1,078 *g* for 10 min, and the absorbance of the supernatant was measured at 532 nm. The MDA content was normalized to the total protein concentration and expressed as a percentage relative to the control group.

### 2.13 Western blot analysis

Cells were lysed in RIPA buffer (Beyotime, China) containing a protease inhibitor cocktail. The lysates were centrifuged at 12,000 × g for 15 min at 4 °C, and the protein concentration in the supernatant was determined using a BCA kit. Equal amounts of protein (20–30 µg) were separated by SDS-PAGE and transferred onto PVDF membranes (Millipore, United States). The membranes were blocked with 5% non-fat milk for 2 h at room temperature and then incubated overnight at 4 °C with primary antibodies against p53, SLC7A11, GPX4, and GAPDH (all from Proteintech Group, United States). After washing, the membranes were incubated with HRP-conjugated secondary antibodies for 2 h at room temperature. Protein bands were visualized using an enhanced chemiluminescence (ECL) detection system, and the band intensities were quantified using ImageJ software.

### 2.14 *In Vivo* xenograft mouse model

All animal experiments were approved by the Animal Experimentation Ethics Committee of Hangzhou City University (Approval No. 23007) and conducted in accordance with the institutional guidelines for the care and use of laboratory animals.

Female BALB/c nude mice (8 weeks old) were purchased from Shanghai Slake Laboratory Animal Co., Ltd. HCT-15 cells (1 × 10^7^) suspended in 100 µL of PBS were subcutaneously injected into the right flank of each mouse. When the tumor volume reached approximately 100 mm^3^, the mice were randomly divided into two groups (n = 6 per group): the control group (administered vehicle) and the acRoots-treated group (administered 150 mg/kg/day via oral gavage). Treatments continued for 14 consecutive days. Body weight and tumor dimensions were measured every 2 days. Tumor volume was calculated using the formula: Volume = (Length × Width^2^)/2. At the end of the experiment, the mice were euthanized, and the tumors were excised and weighed. A portion of each tumor was snap-frozen for GSH analysis.

### 2.15 Statistical analysis

All *in vitro* experiments were performed independently at least three times. Data are presented as the mean ± standard deviation (SD). Statistical analyses were performed using GraphPad Prism software (version 9.3.0). Differences between two groups were analyzed using Student's t-test, and differences among multiple groups were analyzed by one-way analysis of variance (ANOVA). A p-value of less than 0.05 was considered statistically significant.

## 3 Results

We conducted a systematic analysis of the chemical composition of acRoots. Based on previous research, a total of 24 major chemical components were identified from acRoots using ultra-high-performance liquid chromatography coupled with triple quadrupole time-of-flight mass spectrometry (UPLC-Triple-TOF/MS) (Qiu et al., 2023). These components belong to categories such as flavonoids, terpenoids, organic acids, phenols, glycosides, coumarins, and phenylpropanoids, collectively accounting for 15.08% of the total relative peak area. Further screening based on oral bioavailability (OB ≥ 30%), drug-likeness (DL ≥ 0.18), and supporting literature led to the identification of 12 core active compounds, including: procyanidin C1, D-catechin, procyanidin dimer B2, hesperidin, chlorogenic acid, 9,12,13-trihydroxy-10-trans-octadecenoic acid, citric acid, 9(S),12(S),13(S)-trihydroxyoctadeca-10(E),15(Z)-dienoic acid, isochlorogenic acid C, swertiamarin, loganic acid, and fraxetin (Jia et al., 2020; Sung et al., 2013; Villota et al., 2022; Wang et al., 2022; Kim et al., 2023; Chouchkov et al., 1987; Bae et al., 2020), Among these, fraxetin, D-catechin, procyanidin dimer B2, loganic acid, and chlorogenic acid were present at relatively higher levels, while the remaining compounds showed moderate relative contents. It should be noted that the exact mass proportions of individual compounds have not been determined; their relative abundance was established based on chromatographic peak area ratios.

Having established the methodology for preparing and characterizing acRoots, we next sought to systematically evaluate its anti-Colorectal cancer efficacy and mechanism of action. Accordingly, we first determined its effects on fundamental cancer cell behaviors (proliferation, death, migration, and invasion) before focusing on the induction of ferroptosis and the role of the p53/SLC7A11/GPX4 axis. The *in vitro* findings were subsequently corroborated in an *in vivo* setting. The results of this investigation are summarized below.

### 3.1 acRoots suppresses viability, proliferation, and induces cell death in colorectal cancer cells

**FIGURE 1:**
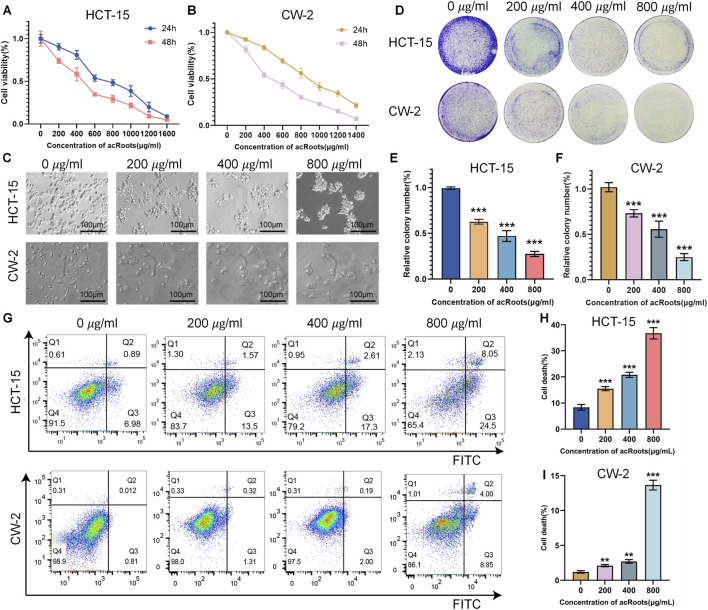
acRoots inhibits viability, proliferation, and induces cell death in Colorectal cancer cells. **(A,B)** Cell viability of HCT-15 and CW-2 cells treated with the indicated concentrations of acRoots for 24 h and 48 h was assessed by CCK-8 assay. **(C)** Representative images showing morphological changes in Colorectal cancer cells after 24 h of acRoots treatment. Scale bar: 10 μm. **(D–F)** Colony formation assays evaluating the long-term proliferative capacity of HCT-15 and CW-2 cells after acRoots treatment. **(G–I)** Cell death was quantified by flow cytometry using Annexin V-FITC/PI staining after 24 h of acRoots treatment. acRoots, *Actinidia chinensis* Planch Root extracts; CCK-8, Cell Counting Kit-8; Colorectal cancer, Colorectal cancer.

To evaluate the cytotoxic effect of acRoots on Colorectal cancer cells, a preliminary experiment was first conducted: HCT-15 and CW-2 cells were treated with a concentration gradient of acRoots ranging from 0 to 1,600 μg/mL. After 24 and 48 h of incubation, cell viability was measured using the CCK-8 assay, and dose-response curves were plotted. The results showed that acRoots significantly inhibited the viability of both Colorectal cancer cell lines in a dose-dependent manner (Figures 1A,B), indicating that its cytotoxicity exhibits clear concentration dependence.

Based on the dose-response curve, four concentrations—0, 200, 400, and 800 μg/mL—were selected for subsequent mechanistic studies. This gradient covers the full range of effects from no obvious inhibition to significant suppression of cell viability, making it suitable for analyzing the relationship between dose and effect in mechanistic exploration. Furthermore, after 24-h treatment with acRoots, distinct morphological changes were observed in the Colorectal cancer cells, including cytoplasmic vacuolization (Figure 1C).

We next evaluated the impact of acRoots on long-term proliferative capacity using a colony formation assay. As shown in Figures 1D–F, acRoots treatment markedly inhibited the colony-forming ability of HCT-15 and CW-2 cells in a dose-dependent manner.

To further investigate cell death induction, we performed Annexin V-FITC/PI staining followed by flow cytometry. Compared to the control, acRoots treatment resulted in a significant increase in the proportion of dead cells (Figures 1G–I). Collectively, these results demonstrate that acRoots exerts potent toxic effects on human Colorectal cancer cells, suppressing proliferation and inducing cell death in a dose-dependent manner.

### 3.2 acRoots impairs the migratory and invasive capacities of colorectal cancer cells

**FIGURE 2:**
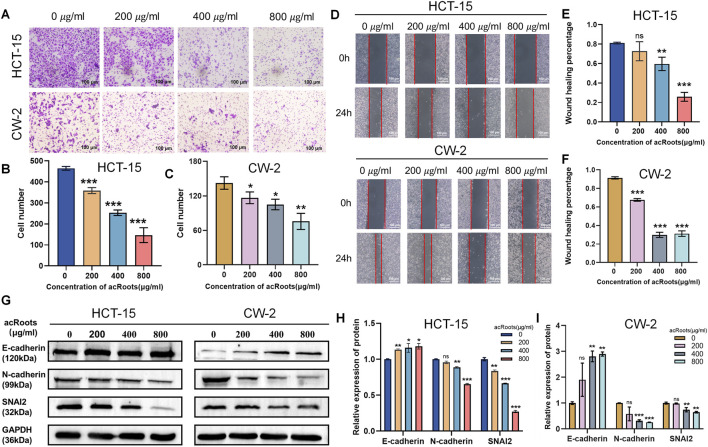
acRoots suppresses the migratory and invasive capacities of Colorectal cancer cells by inhibiting EMT. **(A–C)** Transwell invasion assays showing the number of invading Colorectal cancer cells after acRoots treatment. **(D–F)** Wound healing assays demonstrating the migratory capacity of Colorectal cancer cells after acRoots treatment. **(G–I)** Western blot analysis of EMT markers (E-cadherin, N-cadherin, Vimentin) in Colorectal cancer cells treated with acRoots. Colorectal cancer, Colorectal cancer; acRoots, Actinidia chinensis Planch Root extracts; EMT, Epithelial-Mesenchymal Transition.

The anti-metastatic potential of acRoots was investigated through wound healing and Transwell assays. In the wound healing assay, control cells effectively migrated into the scratched area within 24 h. In contrast, acRoots treatment led to a concentration-dependent inhibition of this migration, as evidenced by a gradual reduction in wound closure (Figures 2D–F).

Similarly, the Transwell assay demonstrated that acRoots significantly diminished the number of cells capable of invading through the Matrigel-coated membrane (Figures 2A–C), indicating a robust suppression of invasive potential.

Since the epithelial-mesenchymal transition (EMT) is a pivotal driver of cancer cell migration and invasion, we examined the expression of key EMT markers. Western blot analysis revealed that acRoots treatment downregulated the mesenchymal markers N-cadherin and vimentin, while upregulating the epithelial marker E-cadherin (Figures 2G–I). This suggests that acRoots attenuates the migratory and invasive phenotypes of Colorectal cancer cells, at least in part, by reversing the EMT process.

### 3.3 acRoots triggers ferroptosis in colorectal cancer cells via ROS accumulation and GSH depletion

Given the observed non-apoptotic morphological changes and the established role of ferroptosis in cancer therapy, we hypothesized that acRoots might induce this form of cell death. Ferroptosis is characterized by the iron-dependent accumulation of lipid-ROS when the GSH-dependent antioxidant defense is compromised.

**FIGURE 3:**
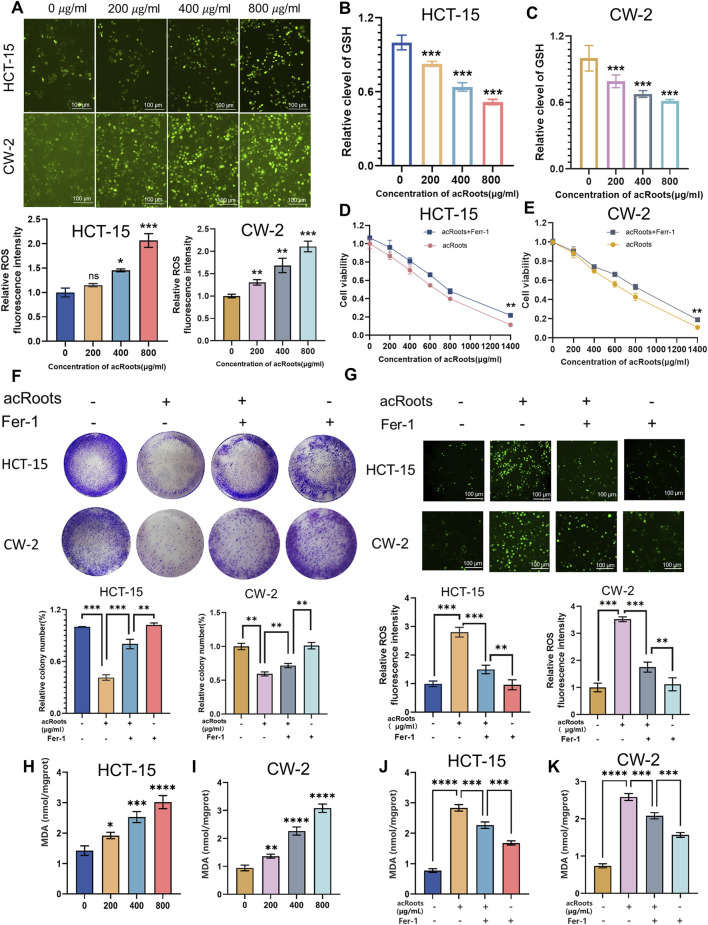
acRoots induces ferroptosis in Colorectal cancer cells. **(A)** Intracellular ROS levels were measured by DCFH-DA fluorescence in Colorectal cancer cells treated with acRoots. **(B,C)** Intracellular GSH levels in acRoots-treated Colorectal cancer cells. **(D,E)** Cell viability was assessed by CCK-8 assay in Colorectal cancer cells pretreated with the ferroptosis inhibitor Fer-1 (1 μM) followed by acRoots. **(F)** Colony formation assay of Colorectal cancer cells pretreated with Fer-1 followed by acRoots. **(G)** Intracellular ROS levels in Colorectal cancer cells pretreated with Fer-1 followed by acRoots. **(H,I)** Intracellular MDA levels in acRoots-treated Colorectal cancer cells. **(J,K)** Intracellular MDA levels in acRoots-treated Colorectal cancer cells pretreated with the ferroptosis inhibitor Fer-1 followed by acRoots. Data are presented as mean ± SD; *, P < 0.05; **, P < 0.01; **, P < 0.001 vs. control group. ROS, reactive oxygen species; CRC, Colorectal cancer; acRoots, Actinidia chinensis Planch Root extracts; GSH, glutathione; CCK-8, Cell Counting Kit-8; Fer-1, Ferrostatin-1.

Using the DCFH-DA fluorescent probe, we found that acRoots treatment induced a pronounced, dose-dependent increase in intracellular ROS levels in both HCT-15 and CW-2 cells (Figure 3A). Concomitantly, acRoots led to a significant depletion of intracellular GSH, a key antioxidant that protects against ferroptosis (Figures 3B,C). To further validate lipid peroxidation—a core marker of ferroptosis—we detected the level of its representative product, MDA, using the colorimetric assay kit. The results showed that acRoots treatment induced a dose-dependent increase in MDA levels in both HCT-15 and CW-2 cells, consistent with the trends observed for ROS accumulation and GSH depletion (Figures 3H,I).

To confirm the involvement of ferroptosis, we employed the specific ferroptosis inhibitor, Fer-1. Pretreatment with Fer-1 significantly rescued acRoots-induced cytotoxicity (Figures 3D,E) and restored the proliferative capacity of the cells (Figure 3F). Accordingly, the elevated ROS levels induced by acRoots were markedly attenuated by Fer-1 (Figure 3G). Notably, pretreatment with Fer-1 also reversed the acRoots-induced accumulation of MDA (Figures 3J,K), providing further evidence that acRoots triggers ferroptosis rather than other forms of cell death. These rescue experiments provide compelling evidence that ferroptosis is a primary mechanism of acRoots-induced cell death in Colorectal cancer. These rescue experiments provide compelling evidence that ferroptosis is a primary mechanism of acRoots-induced cell death in Colorectal cancer.

### 3.4 acRoots activates the p53 pathway to suppress the SLC7A11/GPX4 axis

**FIGURE 4:**
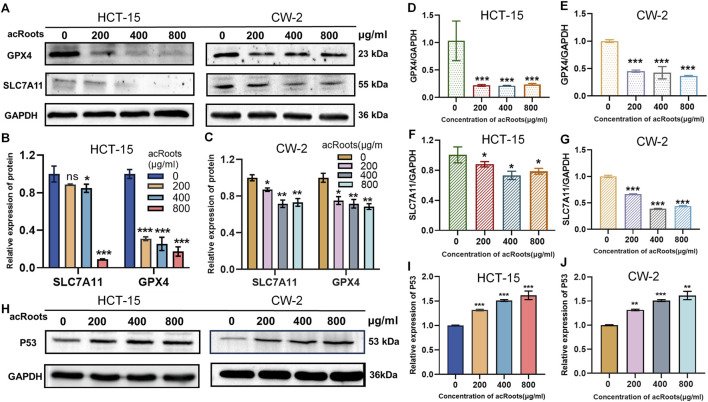
acRoots induces ferroptosis through the p53/SLC7A11/GPX4 axis. **(A–C)** Western blot analysis of GPX4 and SLC7A11 protein expression in Colorectal cancer cells treated with acRoots. **(D,E)** qRT-PCR analysis of GPX4 mRNA expression in acRoots-treated Colorectal cancer cells. **(F,G)** qRT-PCR analysis of SLC7A11 mRNA expression in acRoots-treated Colorectal cancer cells. **(H–J)** Western blot analysis of p53 protein expression in Colorectal cancer cells treated with acRoots. GPX4, glutathione peroxidase 4; SLC7A11, Solute Carrier Family 7 Member 11; Colorectal cancer, Colorectal cancer; acRoots, Actinidia chinensis Planch Root extracts; p53, tumor protein 53.

We next sought to elucidate the molecular pathway underlying acRoots-induced ferroptosis. The SLC7A11/GPX4 axis is a central regulatory node of ferroptosis. Western blot analysis demonstrated that acRoots treatment significantly suppressed the protein expression of both SLC7A11 and GPX4 (Figures 4A–C). Consistent with this, qRT-PCR experiments revealed a concomitant decrease in the mRNA levels of SLC7A11 and GPX4 (Figures 4D–G), indicating transcriptional regulation.

As the tumor suppressor p53 is a known transcriptional repressor of SLC7A11, we investigated its role. Strikingly, acRoots treatment resulted in a substantial upregulation of p53 protein levels in a dose-dependent manner (Figures 4H–J). These results delineate a clear mechanistic pathway wherein acRoots upregulates p53, which is implicated in the transcriptional repression of SLC7A11 and its downstream target GPX4, thereby disabling the cellular defense against lipid peroxidation and committing the cells to ferroptosis.

### 3.5 acRoots suppresses tumor growth and modulates tumor GSH levels in vivo

**FIGURE 5:**
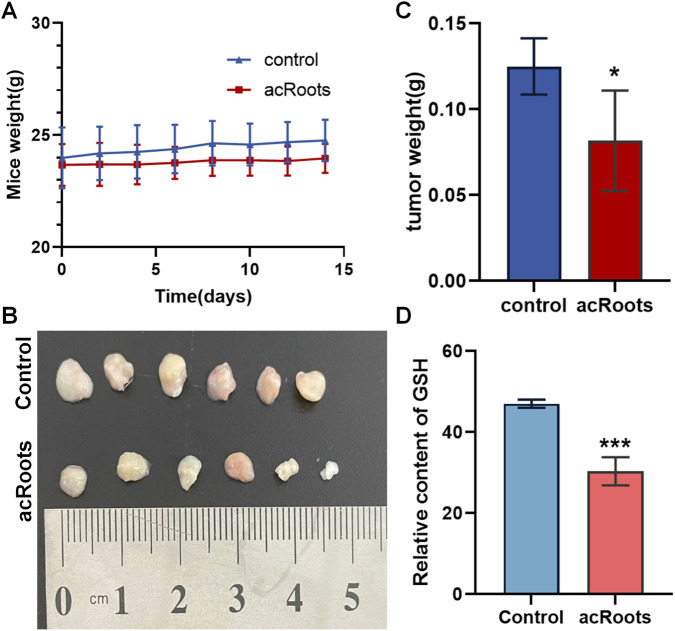
acRoots suppresses tumor growth and depletes GSH in a mouse xenograft model. **(A)** Body weights of mice during the 14-day treatment with acRoots (150 mg/kg/day) or vehicle control. **(B,C)** Tumor size and weight from HCT-15 xenograft mice after acRoots treatment. **(D)** GSH content in tumor tissues from the control and acRoots-treated groups. GSH, glutathione; acRoots, Actinidia chinensis Planch Root extracts.

The antitumor efficacy of acRoots was further validated in a HCT-15 xenograft mouse model. Mice were treated orally with acRoots (150 mg/kg/day) or vehicle control for 14 days. The administration of acRoots did not significantly affect the body weight of the mice (Figure 5A), suggesting a favorable safety profile at this dosage.

Notably, acRoots treatment resulted in a remarkable reduction in both tumor weight and size compared to the control group (Figures 5B,C), corroborating our *in vitro* findings. Furthermore, analysis of tumor tissues revealed a significant decrease in GSH content in the acRoots-treated group (Figure 5D), indicating that the ferroptosis-related mechanism observed *in vitro* is also operative *in vivo*.

## 4 Discussion

Our study demonstrates that acRoots, a TCM, exerts potent anti-tumor effects against Colorectal cancer both *in vitro* and *in vivo*. We provide compelling evidence that the primary mechanism underlying this activity is the induction of ferroptosis, a form of regulated cell death driven by iron-dependent lipid peroxidation. Crucially, we delineate the molecular pathway through which acRoots acts: it upregulates the tumor suppressor p53, leading to the subsequent transcriptional and translational downregulation of the key ferroptosis regulators SLC7A11 and GPX4. This work not only confirms the anti-Colorectal cancer potential of acRoots but also makes it as a novel natural inducer of ferroptosis, offering a good perspective on its therapeutic application. It should be noted that the relationship between the effective concentration *in vitro* (800 μg/mL) and potential safety in humans requires careful consideration of the differences between experimental conditions and the physiological *in vivo* environment. A significant gap between *in vitro* and *in vivo*/clinical doses is common in preclinical research, primarily due to fundamental differences in the setting of action: *in vitro*, the compound acts directly on cells, whereas *in vivo* administration involves complex pharmacokinetic processes such as absorption, distribution, metabolism, and excretion, which significantly influence the actual exposure concentration at the target site (Shan et al., 2016).

Notably, our *in vivo* results demonstrate that in an HCT-15 xenograft mouse model, a dose of acRoots far lower than the *in vitro* concentration (150 mg/kg/day, oral gavage) significantly inhibited tumor growth without observable apparent toxicity (Figure 5A). These findings better reflect the potential therapeutic window and safety profile of acRoots, as they incorporate the complete pharmacokinetic process and tissue distribution effects.

It is important to emphasize that this study represents preclinical mechanistic exploration. The selection of an 800 μg/mL dose *in vitro* was primarily aimed at elucidating its anti-Colorectal cancer mechanism of action and does not directly correspond to a clinical dosing regimen. Determining the human equivalent dose will require further systematic pharmacokinetic and toxicological studies, including measuring plasma concentration-time profiles in animal models, investigating tissue distribution characteristics, and assessing long-term safety. Such work will lay the necessary foundation for rationally designing dosing regimens in future clinical trials.

The cytotoxic, anti-proliferative, anti-migratory, and anti-invasive effects of acRoots observed in our study (Figures 1, 2) are consistent with its reported efficacy in other malignancies, such as gastric and liver cancer (Gao et al., 2020; Fang et al., 2019). While our initial Annexin V/PI staining indicated acRoots-induced cell death (Figures 1G–I), the subsequent rescue experiments with Fer-1, a specific ferroptosis inhibitor, fundamentally shifted our understanding. The fact that Fer-1 significantly reversed the cytotoxic effects and ROS accumulation (Figures 3D–G) strongly suggests that ferroptosis, not apoptosis, is the predominant mode of cell death triggered by acRoots in Colorectal cancer cells. This distinction is critical, as it highlights a unique mechanism that may help overcome apoptosis resistance, a common challenge in cancer therapy.

The core of our mechanistic discovery lies in the acRoots-p53-SLC7A11/GPX4 axis. The observed depletion of GSH and accumulation of ROS (Figures 3A–C) are hallmark biochemical events of ferroptosis. Concurrently, using the MDA colorimetric assay kit, we found that the content of the lipid peroxidation product MDA was significantly increased in a dose-dependent manner following acRoots treatment (Figures 3H,I). This increase could be reversed by the ferroptosis inhibitor Fer-1 (Figures 3J,K), a process directly linked to the function of the system Xc^−^ transporter SLC7A11 and the peroxidase GPX4 (21). Our data clearly show that acRoots suppresses both the mRNA and protein expression of SLC7A11 and GPX4 (Figures 4A–G). This inhibition disrupts cellular antioxidant defense, permitting the lethal accumulation of lipid peroxides. Furthermore, we identified that this suppression is mediated through the significant upregulation of p53 (Figures 4H–J). This finding aligns with the established role of nuclear p53 in repressing SLC7A11 transcription, thereby sensitizing cells to ferroptosis (Xie et al., 2017). Therefore, our study elucidates a coherent pathway: acRoots → p53 ↑ → SLC7A11/GPX4↓→ GSH depletion/ROS accumulation/MDA elevation→ Ferroptosis↑ → SLC7A11/GPX4 ↓ → GSH depletion/ROS accumulation/MDA elevation→ Ferroptosis.

The multi-target nature of TCM extracts suggests that the ferroptosis-inducing effect of acRoots is likely attributable to a constellation of its active metabolites. Previous phytochemical analysis of acRoots identified several metabolites, such as fraxin and chlorogenic acid (Qiu et al., 2023). Intriguingly, chlorogenic acid-derived materials have been reported to exhibit GSH oxidase-like activity and promote ferroptosis (Yao et al., 2022), while fraxin is known to modulate GSH levels (Li et al., 2019). We therefore hypothesize that the collective action of these and other unidentified metabolites in acRoots concurrently targets the intracellular redox homeostasis, culminating in a robust ferroptotic response. This synergistic multi-metabolite action warrants further investigation to pinpoint the most critical bioactive molecules.

Looking forward, the potential of acRoots in clinical application may be maximized through combination strategies. Previous studies have shown that isolated metabolites from acRoots, like ACPS-R and ursolic acid, can enhance the efficacy of conventional chemotherapeutics like 5-FU (Shan et al., 2016; Lin, 1988). Building on this, a key future direction will be to explore the synergy between acRoots and standard Colorectal cancer treatments (e.g., 5-FU, oxaliplatin, or targeted therapies). Such combinations could potentially lower the required doses of cytotoxic chemotherapeutics, reduce side effects, and overcome drug resistance by activating a complementary cell death pathway. Notably, in addition to the combination of traditional chemotherapy and herbal medicine, the potential value of GLP-1 receptor agonists in Colorectal cancer treatment has gradually gained attention in recent years. Studies indicate that GLP-1 receptor agonists can significantly influence the biological behavior of Colorectal cancer cells—including inhibiting proliferation, inducing cell cycle arrest, and reducing invasion and metastasis—by modulating the PI3K/AKT/mTOR signaling pathway (Tong et al., 2022). The PI3K/AKT/mTOR pathway intersects with the p53/SLC7A11/GPX4 axis examined in this study, both of which are involved in regulating cellular redox balance, proliferation, and death.

Future research could explore the potential of combining acRoots with GLP-1 receptor agonists. On one hand, acRoots exerts antitumor effects by inducing ferroptosis; on the other hand, GLP-1 receptor agonists suppress malignant phenotypes of cancer cells through regulation of the PI3K/AKT/mTOR pathway. The two may complement each other mechanistically to enhance anti-Colorectal cancer efficacy, offering a novel combination strategy for clinical treatment.

Despite the compelling evidence, our study has several limitations. First, the necessity of p53 in acRoots-induced ferroptosis, while strongly suggested by our data, requires direct validation in p53-deficient or p53-mutated Colorectal cancer cell models. Second, the precise bioactive metabolite(s) within acRoots responsible for activating the p53 pathway remain to be isolated and identified. Third, our *in vivo* validation was conducted using a single cell line xenograft model; the efficacy of acRoots across different Colorectal cancer subtypes with varying genetic backgrounds needs further exploration. Fourth, although our data strongly suggest a close association between p53 activation and acRoots-induced ferroptosis, functional loss-of-function validation using p53 inhibitors or p53-knockout cell models has not been conducted. Therefore, the conclusion that “p53 is involved in mediating acRoots-induced ferroptosis” remains based on strong correlative evidence rather than direct proof of p53’s necessity. Furthermore, although *in vivo* experiments demonstrated that acRoots reduces intratumoral GSH levels and exerts antitumor effects, this study did not evaluate the expression and localization of p53, SLC7A11, and GPX4 in tumor tissues through immunohistochemical (IHC) or immunofluorescence (IF) staining. Consequently, direct morphological evidence of the regulatory role of this pathway *in vivo* is lacking. To address this limitation, subsequent research will incorporate IHC/IF staining techniques to clarify the expression and spatial distribution of key molecules within the p53/SLC7A11/GPX4 axis in tumor tissues. Subsequent research will employ genetically modified cell models (e.g., p53-knockout cells) and p53 inhibitors to directly determine whether p53 is essential for acRoots-induced ferroptosis. Fifth, we recognize the present study used UPLC-Triple-TOF/MS high-resolution mass spectrometry. This technique systematically identified the major chemical components in acRoots. It provided critical information, including chromatographic retention times, accurate mass values, and fragment ions. These data establish an important basis for understanding the material composition of the extract. However, relying on a single analytical method has limitations. Its comprehensiveness for full chemical characterization remains constrained. To meet the requirements for future standardized drug development, subsequent research will integrate multiple orthogonal analytical techniques (e.g., HPLC-UV, HPLC-ELSD) to establish a more complete chemical fingerprint profile. This will enable a systematic evaluation of batch-to-batch consistency and quality controllability, thereby providing more robust and comprehensive pharmaceutical data to support the translational development of this extract toward clinical application. Future clinical trials must also carefully evaluate the pharmacokinetics, safety, and optimal dosing of acRoots in diverse patient populations to fully assess its translational potential.

In conclusion, our findings establish that acRoots is an effective ferroptosis inducer in Colorectal cancer. By unveiling the pivotal role of the p53-SLC7A11/GPX4 signaling axis in this process, we provide a solid mechanistic foundation for its anti-tumor activity, though the necessity of p53 requires further validation with functional experiments. These insights not only enrich our understanding of this TCM but also advocate for its continued development as a promising therapeutic agent or adjuvant for Colorectal cancer treatment.

## Data Availability

The raw data supporting the conclusions of this article will be made available by the authors, without undue reservation.

## Supplementary material

SUPPLEMENTARY FIGURE S1 Base peak chromatogram of acRoots by UPLC-Triple-TOF/MS.

SUPPLEMENTARY TABLE S1 Main Chemical Constituents of acRoots.

SUPPLEMENTARY TABLE S2 The PCR primers. GPX4, glutathione peroxidase 4; GAPDH, Glyceraldehyde-3-phosphate dehydrogenase; SLC7A11, Solute Carrier Family 7 Member 11.
