# Supplementary figures and images for: Actinidia chinensis Planch Root extracts trigger ferroptosis in colorectal cancer via the p53/SLC7A11/GPX4 axis
Source: Front Pharmacol. 2026 Jan 14;17:1724983. doi: 10.3389/fphar.2026.1724983 (PMC12847262; doi:10.3389/fphar.2026.1724983)

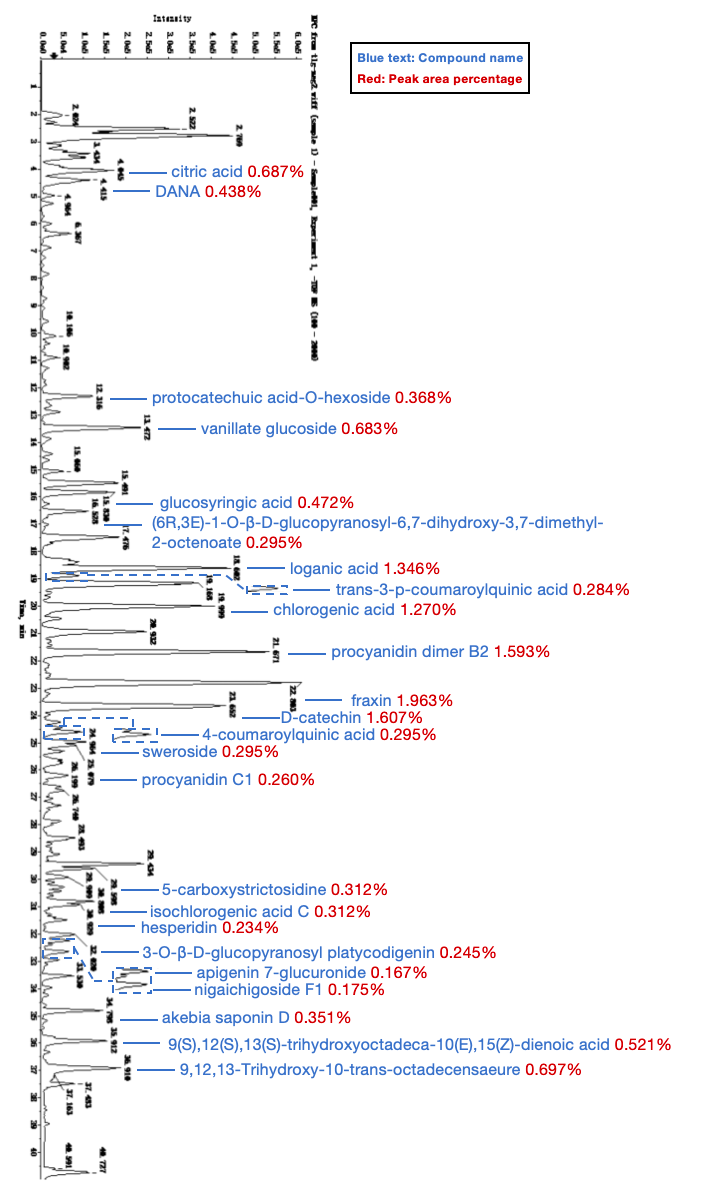

Supplement: Supplementary file 2 [file Image1.tif]
